# Fosfomycin in continuous or prolonged infusion for systemic bacterial infections: a systematic review of its dosing regimen proposal from in vitro, in vivo and clinical studies

**DOI:** 10.1007/s10096-021-04181-x

**Published:** 2021-02-18

**Authors:** Roberta Maria Antonello, Stefano Di Bella, Alberto Enrico Maraolo, Roberto Luzzati

**Affiliations:** 1 Clinical Department of Medical, Surgical and Health Sciences, Trieste University, 34127 Trieste, Italy; 2 First Division of Infectious Diseases, Cotugno Hospital, AORN dei Colli, 80131 Naples, Italy

**Keywords:** Fosfomycin, Continuous infusion, Prolonged infusion, Pharmacokinetics, Pharmacodynamics, Infection

## Abstract

Fosfomycin (FOS) administered intravenously has been recently rediscovered for the treatment of systemic infections due to multidrug-resistant bacteria. Its pharmacokinetic properties suggest a time-dependent dosing schedule with more clinical benefits from prolonged (PI) or continuous infusion (CI) than from intermittent infusion. We revised literature concerning PI and CI FOS to identify the best dosing regimen based on current evidence. We performed a MEDLINE/PubMed search. Ninety-one studies and their pertinent references were screened. Seventeen studies were included in the present review. The activity of FOS against Gram-negative and Gram-positive bacteria was evaluated in fourteen and five studies, respectively. Six studies evaluated FOS activity in combination with another antibiotic. Daily dosing of 12, 16, 18 or 24 g, administered with different schedules, were investigated. These regimens resulted active against the tested isolates in most cases. Emergence of resistant isolates has been shown to be preventable through the coadministration of another active antibiotic. FOS is a promising option to treat systemic infections caused by multidrug-resistant bacteria. Coadministration with another active molecule is required to prevent the emergence of resistant bacterial strains. The results of our review suggest that a therapeutic regimen including a loading dose of FOS 8 g followed by a daily dose of 16 g or 24 g CI could be the best therapeutic approach for patients with normal renal function. The dosing regimens in patients with renal insufficiency and CI or PI superiority compared with intermittent infusion in clinical settings should be further investigated.

## Introduction

The worrying increase of antimicrobial resistance, both in inpatients and outpatients, prompts clinicians to find new therapeutic options. Fosfomycin (FOS), administered intravenously, has been recently re-evaluated for the treatment of systemic infections caused by multidrug-resistant (MDR) bacteria. FOS acts with a unique mechanism of action on the bacterial wall. It is active against many aerobic Gram-negative and -positive bacterial strains (Table 1) [2], and it should be administered with (at least) another active drug to prevent the emergence of resistance [3, 4].

**Table 1:** Aerobic Gram-positive and Gram-negative strains susceptible to fosfomycin [1].

Aerobic gram-positive cocci	Aerobic GNB (*Enterobacterales*)	Aerobic GNB—selected non-fermentative
*Enterococcus* spp. (also VRE)	*E. coli* (+ ESBL and KPC* producers)	P. aeruginosa*
*S. aureus* (also MRSA)	*Klebsiella* spp. (+ ESBL and KPC* producers)	
*Staphylococcus* spp. coagulase-negative	*Citrobacter* spp.	
*S. lugdunensis*	*Enterobacter* spp.	
S. saprophyticus*	*P. vulgaris*^a^	
	*Serratia* spp.	

*Weak activity

GNB, Gram-negative bacilli; VRE, vancomycin-resistant enterococci; MRSA, methicillin-resistant *S. aureus*; KPC, *K. pneumoniae* carbapenemase; ESBL, extended-spectrum beta-lactamase

FOS is marketed both as oral (fosfomycin trometamol, fosfomycin calcium) and intravenous (fosfomycin disodium) formulation. Both time- and concentration-dependent activity have been suggested according to the bacteria evaluated, but due to its short half-life and rapid bactericidal action a time-dependent approach is more often employed [3, 5, 6]. FOS serum half-life is 4–5.7 h for oral formulation [7, 8] and approximately halved when administered intravenously [8, 9]. Although literature data on FOS volume of distribution are controversial (ranging from 40 to 136 L [6, 10]), an excellent tissue penetration is reported, including the central nervous system, soft tissues and bone tissues [6] (AUC_0–4_ ratio for muscle over plasma was 0.71 for patients with soft tissue infections [11]). FOS is an appealing therapeutic option also for lower respiratory tract infections, biliary tract infections and abscesses [12–15]. FOS is cleared non-metabolized by the kidney and reaches in urine concentrations higher than the minimum inhibitory concentrations (MICs) [8, 16, 17]. Urinary concentrations are higher when FOS is administered intravenously [8]. Its pharmacokinetic properties suggest a time-dependent dosing schedule, with potential clinical benefits deriving from prolonged (PI) or continuous infusion (CI) compared with intermittent infusion (II), the dosing schedule most frequently used to-date. Despite this, guidelines on the best dosing regimen for FOS are lacking. Therefore, we revised literature concerning FOS CI or PI to hypothesize the best dosing regimen based on the actual evidence.

## Materials and methods

We performed a MEDLINE/PubMed search and the complete search string was as follows: “(fosfomycin[Text Word]) AND (continuous[Text Word] OR prolonged[Text Word] OR extended[Text Word]) AND (infusion[Text Word] OR intravenous[Text Word] OR pharmacodynamics[Text Word]OR pharmacokinetics[Text Word] OR “opat”[Text Word] OR outpatient[Text Word] OR elastomeric[Text Word] OR pump[Text Word])”. Ninety-one papers from inception to 4 November 2020 were identified and underwent title, abstract and full text screening. Papers written in languages other than English were excluded. Seventy-six papers were excluded for the aforementioned reasons. In addition, pertinent references of included papers and abstracts from international congresses (from 2016 to 2020) were reviewed and discussed. A total of seventeen papers were included in the present review.

## Results

Seventeen papers (14 original articles, 4 of which clinical trials, 2 abstracts from international congress and 1 review) were reviewed and discussed. Preclinical and clinical studies evaluated in the present review are briefly listed in Table 2 and Table 3, respectively.

**Table 2:** Review of literature concerning FOS in continuous or prolonged infusion (preclinical studies).

Author, country and year	Type of paper	Methods	Bacteria (number)	Combination or comparison with	FOS dosing regimens	Comments
Guggenbichler et al. 1992 [18]	Original article	Catheter infection model; catheter sepsis (5)	*S. aureus* (1), *S. epidermidis* (1)	-	24-, 48- or 96-hr CI at a concentration of 100 μg/mL (flow rate 20 mL/hr)	Combination of FOS CI and imipenem/cilastatin resulted in microbiological and clinical success in 5 out of 5 episodes (*S. epidermidis*).
Chavanet et al. 1995 [19]	Original article	Fibrin clots infection model	*S. pneumoniae* (1)	Cefotaxime	FOS monotherapy 6-hr CI including a 25 mg/kg LD followed by 75 mg/kg FOS + CTX FOS 6-hr CI including a 25 mg/kg LD followed by 75 mg/kg + CTX 25 mg/kg LD followed by 75 mg/kg	The authors also evaluated single-dose FOS and CTX, alone and in combination, and this resulted in a higher AUC compared with CI.
Xiong et al. 1995 [20]	Original article	Rabbit endocarditis model	*P. aeruginosa* (2)	Ciprofloxacin, pefloxacin	FOS + CIP FOS 300 mg/kg 24-hr CI + CIP 64 mg/kg 24-hr CI FOS + PEF FOS 300 mg/kg 24-hr CI + PEF 64 mg/kg 24-hr CI	Additive and synergistic effect was observed for the combinations FOS + CIP and FOS + PEF, respectively. FOS + CIP lead to a greater reduction in the number of CFU per gram of vegetations.
Bugnon et al. 1997 [21]	Original article	Rabbit endocarditis model	*P. aeruginosa* (2)	-	300 mg/kg/day CI	Compared with pefloxacin, FOS had a greater and more constant bactericidal effect.
Docobo-Pérez et al. 2015 [22]	Original article	Hollow fibre infection model	ESBL-producing *E. coli* (3)	Meropenem	FOS MIC ≤ 1 mg/L FOS monotherapy 12 g CI FOS + MEM Not evaluated as continuous infusion. FOS 4 g q8hr + MEM 1 g q8hr	For one isolate even higher dosages of FOS monotherapy (24g q24hr; 36 g q24hr) were ineffective due to selection of resistant mutants.
Asuphon et al. 2016 [23]	Original article	Monte Carlo simulation	*P. aeruginosa* (120)	Carbapenems	Non-MDR isolates FOS monotherapy 4 g q8hr PI (above 90% PTA) FOS + carbapenems FOS 16 g CI + MEM 1–2 g q8hr PI (80% PTA) FOS 16 g CI + DOM 1 g q8hr PI (80% PTA) MDR isolates FOS monotherapy 4 g q4hr PI (above 90% PTA) FOS + carbapenems All combinations could not achieve the PK/PD targets against MDR PA. FOS 8 g q8hr PI + DOM 1 g q8hr PI achieved the target against CRPA.	Prolonged and continuous infusions improved PK/PD exposure compared with dosage regimens using traditional 30-min infusions.
Albiero et al. 2016 [24]	Original article	Monte Carlo simulation	KPC-producing *K. pneumoniae* (18)	Meropenem	FOS monotherapy - FOS + MEM FOS 6 g q6hr PI + MEM 1.5 g q6hr PI (80–90% PTA) FOS 8 g q8hr PI + MEM 1.5 g q6hr PI (80–90% PTA)	Data were simulated for patients with normal renal function. For patients with renal impairment, percentages of PTA are higher (FOS monotherapy 6 g q6hr PI or 8 g q8hr PI above 90%). In case of MEM MICs ≥ 32 mg/L, none of the dosing regimens of MEM reached 90% PTA.
Bhavnani et al. 2017 [25]	Abstract	PK model simulation	*Enterobacterales* (considered for their representative MICs)	-	FOS MIC ≤ 64 mg/L, ClCr ≥ 50 mL/min/1.73m^2^ 6 g q8hr PI (> 90% PTA)	-
Louie et al. 2018 [4]	Original article	Hollow fibre infection model	*P. aeruginosa* (1)	-	12 g CI or 18 g CI (see Comments).	All FOS regimens rapidly selected for resistant isolates, irrespective of the dose or fractionation schedule. With CI (12 g CI or 18 g CI) regimens, resistance emerged later (6 hr vs. 4 hr).
Diep et al. 2018 [26]	Original article	Hollow fibre infection model	*K. pneumoniae* KPC-producing (2)	Polymyxin B	FOS monotherapy 6 g q6hr PI (1-hr or 3-hr infusion) Rapid bactericidal effect, followed by regrowth after 24 hr FOS + PMB FOS 6 g q6hr PI (1-hr or 3-hr infusion) + PMB 2.5-mg/kg LD (2-hr infusion) followed by 1.5 mg/kg q12hr (1-hr infusion)	The combination of FOS and PMB had a synergistic effect with sustained bactericidal effect.
Rodrìguez-Gascón et al. 2019 [27]	Review	Revision of literature	Comparison with MICs for *Enterobacterales*, *P. aeruginosa* and *Staphylococcus* spp.	-	FOS MIC ≤ 64 mg/L 6 g q 6hr PI (30 min) 8 g q 8hr PI (30 min or 6 hr)	FOS monotherapy was not able to achieve PK/PD targets for strains of MIC ≥ 128 mg/L.
Leelawattanachai et al. 2020 [28]	Original article	Monte Carlo simulation	Carbapenem-resistant *Enterobacterales*: 116 *K. pneumoniae*, 12 *E. coli*, 1 *E. cloacae*	-	FOS MIC ≤ 128 mg/L, weight 50 kg, ClCr ≥ 80 mL/min/1.73m^2^ 8 g q8hr PI (90% PTA) 8 g q12hr PI (90% PTA) 4 g q6hr PI (90% PTA)	-

FOS, fosfomycin; CTX, cefotaxime; CIP, ciprofloxacin; PEF, pefloxacin; MEM, meropenem; DOM, doripenem; PMB, polymyxin B; CI, continuous infusion; PI, prolonged infusion; LD, loading dose; MIC, minimum inhibitory concentration; MDR, multidrug-resistant; PK, pharmacokinetics; PD, pharmacodynamics; PTA, probability of target attainment; AUC, area under the curve; ClCr, creatinine clearance; CRPA, carbapenem-resistant *P. aeruginosa*; ESBL, extended-spectrum beta-lactamase; KPC, *K. pneumoniae* carbapenemase

**Table 3:** Review of literature concerning FOS in continuous or prolonged infusion (clinical studies).

Author, country and year	Type of paper	Methods	Bacteria (number)	Combination or comparison with	FOS dosing regimens	Comments
Merino-Bohórquez et al. 2018 [29]	Original article (clinical trial)	Bacteraemic UTI; Monte Carlo simulation	MDR *E. coli* (16)	-	4 g q6hr PI (non-superior: 8 g q8hr PI)	Decrease 1-log bacterial burden in 89–96% (EUCAST breakpoints) and 33–54% (CLSI breakpoints) of patients.
Matzneller et al. 2019 [30]	Abstract	Clinical (healthy volunteers)	P. aeruginosa*	-	1 g/hr CI preceded by a LD of 8 g over 30 min	CI resulted in 100% PTA for MICs up to 128 mg/L. Intermittent intravenous infusion resulted in markedly lower % PTA.
Eckburg et al. 2017 [31] Kaye et al. 2019 [32]	Original article (clinical trial)	184 hospitalized patients with complicated UTI or acute pyelonephritis (+ 231 treated with piperacillin-tazobactam)	*Enterobacterales*, *P. aeruginosa*, *A. baumannii-calcoaceticus* complex, *E. faecalis*, *S. aureus*, *S. saprophyticus*	-	ClCr ≥ 20 mL/min/1.73 m^2^ 6 g q8hr PI	FOS resulted as non-inferior to piperacillin-tazobactam. FOS resulted in overall success rate of 64.7% (119/184 patients). PIP/TAZ resulted in overall success rate of 54.5% (97/178 patients).
Al Jalali et al. 2020 [33]	Original article (clinical trial)	Randomized crossover study in 8 healthy volunteers	- (PK/PD study)	-	8 g over 30 min LD + 1 g/hr CI	Comparison with intermittent infusion 8 g over 30 min every 8 hr showed better PK/PD parameters in volunteers who received CI.

*The study was conducted on healthy volunteers and data obtained were compared with representative MICs of *P. aeruginosa* isolates

FOS, fosfomycin; PIP/TAZ, piperacillin/tazobactam; CI, continuous infusion; PI, prolonged infusion; LD, loading dose; MIC, minimum inhibitory concentration; MDR, multidrug-resistant; PK, pharmacokinetics; PD, pharmacodynamics; ClCr, creatinine clearance; UTI, urinary tract infection

Fourteen studies investigated FOS dosing regimens in the setting of Gram-negative bacteria (2 *in vivo* studies, 8 simulation studies, 4 clinical trials, 1 review [4, 20–32]), while FOS dosing regimens against Gram-positive bacteria were evaluated in 5 studies (2 *in vitro* studies, 2 clinical trials, 1 review [18, 19, 27, 31, 32]). One study [33] did not evaluate the activity of FOS administered in CI since its objective was to report PK/PD parameters in healthy volunteers. Six studies [19, 20, 22–24, 26] evaluated FOS in combination with cefotaxime, ciprofloxacin, pefloxacin, meropenem, doripenem and polymyxin B.

With regard to CI, the daily dosing regimens in the setting of FOS monotherapy were 12 g [4, 22], 18 g [4] and 24 g [30, 33], while FOS in combination with carbapenems was evaluated at daily dose of 16 g [23], resulting active against *Pseudomonas aeruginosa* in two studies [23, 30] and *Escherichia coli* extended-spectrum beta-lactamase (ESBL)-producing, but not against carbapenem-resistant *P. aeruginosa*.

With regard to PI, seven different dosing regimens were evaluated. A schedule of 12 g per day (4g q8hr PI) was evaluated in two studies against non-MDR isolates, administered as monotherapy [23] or combination therapy [22]. FOS monotherapy 16 g per day, administered either as 4 g q6hr PI [28, 29] or 8 g q12hr PI [28], resulted active against non-MDR isolates in two studies. Administration of 18 g per day (6 g q8hr PI) was evaluated in a PK model simulation [25] and in the ZEUS trial [31, 32]. Finally, dosing regimens of 24 g per day, either as 4 g q4hr PI [23], 6 g q6hr PI [24, 26, 27], or 8 g q8hr PI [24, 27–29], resulted active also against MDR isolates.

When FOS given as monotherapy did not result to be active, this was due to the emergence of resistant strains [4, 22, 26]. FOS resistance occurred later when FOS was administered in CI compared with intermittent infusion [23, 26]. The administration of FOS with another active antibiotic was able to overcome resistance in most cases obtaining sustained bactericidal effect [23, 26].

PI resulted in 80–90% probability of target attainment (PTA) in studies simulating the efficacy of FOS against both *P. aeruginosa* and *Enterobacterales* [23–25, 28]. FOS administered in CI showed even better results, reaching 100% PTA against *P. aeruginosa* isolates in the study by Matzneller *et al.* [30]*.*

Table 4 sums up the investigated dosing regimens and their effectiveness against the tested isolates.

**Table 4:**
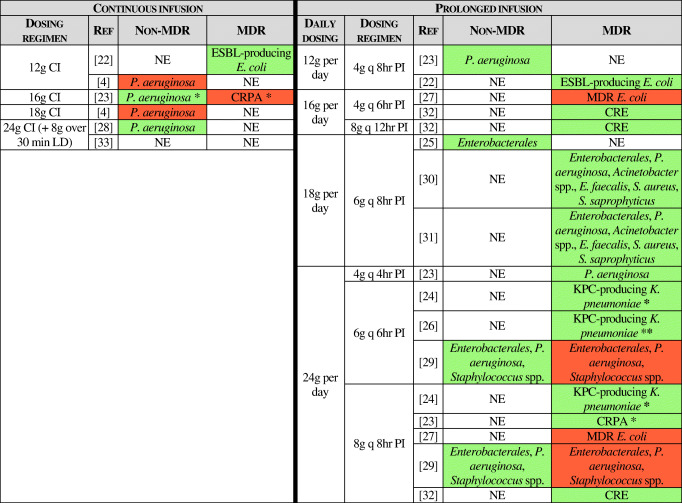
FOS administered as continuous or prolonged infusion: dosing regimens evaluated in the reviewed studies. Dosing regimens active against the tested isolates are highlighted in green, while ineffective regimens are presented in red. NE, not evaluated; CI, continuous infusion; PI, prolonged infusion; LD, loading dose; REF, reference; ESBL, extended-spectrum beta-lactamase; KPC, *K. pneumoniae* carbapenemase; CRPA, carbapenem-resistant *P. aeruginosa*; CRE, carbapenem-resistant *Enterobacterales*. *In combination with carbapenems. **In combination with polymyxin B

## Discussion and conclusion

This is the first systematic review evaluating FOS administered as CI or PI. Actual guidelines or expert opinions indicate slightly different dosages for the administration of FOS in CI [34, 35].

Our revision suggests that FOS 8 g loading dose followed by a daily dose of 16 g or up to 24 g CI is the best approach for patients with normal renal function. This dosage should be tailored considering the site of infection and the FOS MIC of the bacteria responsible of the infection. A critical evaluation of different dosing regimens should always be performed. For instance, evaluation of FOS penetration in abscesses reported a long half-life of the molecule (32 ± 39 h) in the pus, suggesting that FOS CI would not add any advantage compared with II in this scenario [36]. This is due to the fact that CI leads to higher AUC but reduced *C*_max_ compared with II [30, 33].

FOS administered according to dosing regimens CI or PI is an option to keep in mind to treat systemic infections caused by MDR bacteria. Although FOS turned out to be well tolerated, thrombophlebitis and circumscribed paresthesia were reported to occur especially when the antibiotic is administered according to the CI or PI regimens [30, 33]. Administration of IV Ringer’s lactate simultaneously with FOS reduced the risk of thrombophlebitis in one study [33].

Dose adjustment according to renal function is required to keep the good safety profile of the drug, as acute or chronic kidney injury can cause a reduction in the glomerular filtration and therefore in the drug elimination [37, 38].

The emergence of resistant bacterial strains resulted in a weak activity of FOS in some series [4, 22, 26]. About this critical issue, CI delayed the development of resistance to FOS compared with II [4]. FOS has excellent synergistic properties [39] and these can lead to a long-lasting bactericidal effect [23, 26]. Furthermore, taking the advantages obtained by the synergism of FOS with other antibiotics, FOS can be considered for the combination treatment of some isolates intrinsically resistant to FOS or against which FOS has only a weak activity, i.e., *P. aeruginosa* or *Acinetobacter* spp. [23, 40–43]. Indeed, FOS represents a good option for combination therapies with antibiotics active against such bacteria.

Another advantage of PI or CI is the potential decrease of electrolyte imbalance if compared with rapid infusion [44]. In fact, the intravenous formulation contains 13.5 mEq/g of sodium; therefore, caution is needed to avoid hyopokalemia, especially in patients with heart insufficiency or who are undergoing dialysis [34].

Although few clinical studies evaluating FOS in CI or PI against Gram-positive bacteria are available to-date, this review suggests potential benefits from the use of this antibiotic in this setting [18, 19, 27, 31, 32]. This is interesting if we take into consideration the anti-biofilm properties of FOS, against both Gram-positive and Gram-negative bacterial strains [45, 46].

To the best of our knowledge, no study evaluated the efficacy of FOS prescribed as CI in outpatients through elastomeric pumps. Due to its long-term stability, intravenous FOS CI might be an option also for the outpatient parenteral antimicrobial therapy (OPAT), thus shortening hospitalization and its related risks and costs.

In summary, this systematic review suggests that FOS 8 g loading dose followed by a daily dose of 16 g or up to 24 g CI is a promising therapeutic regimen in the treatment of systemic infections including those due to MDR organisms. Future studies on FOS administered according to the CI regimen should include the evaluation of dosing regimens in patients with chronic renal failure and in haemodialysed patients. The efficacy of FOS according to the site of infection requires further investigation and expert advice should always be sought. Furthermore, as evaluation of PK/PD parameters on healthy volunteers after CI showed better results compared with II [30, 33], clinical trials comparing the superiority of CI or PI to II in different settings are desirable.
